# Comparison of P-wave dispersion in healthy dogs, dogs with chronic valvular disease and dogs with disturbances of supraventricular conduction

**DOI:** 10.1186/1751-0147-53-18

**Published:** 2011-03-11

**Authors:** Agnieszka Noszczyk-Nowak, Anna Szałas, Urszula Pasławska, Józef Nicpoń

**Affiliations:** 1 Department of Internal Diseases with Clinic for Horses, Dogs and Cats, Faculty of Veterinary Medicine, Wrocław University Of Environmental And Life Sciences, Grunwaldzki sq. 47, 50-366 Wrocław, Poland

## Abstract

**Background:**

P-wave dispersion (P_d_) is a new ECG index used in human cardiology and veterinary medicine. It is defined as the difference between the maximum and the minimum P-wave duration recorded from multiple different ECG leads. So far no studies were performed assessing the importance of P-wave dispersion in dogs.

**Methods:**

The current study was aimed at determining proper value of P_d _in healthy dogs (group I), dogs with chronic valvular disease (group II) and dogs with disturbances of supraventricular conduction (group III). The tests were carried out in 53 healthy dogs, 23 dogs with chronic valvular disease and 12 dogs with disturbances of supraventricular conduction of various breeds, sexes and body weight from 1,5 to 80 kg, aged between 0,5 and 17 years, submitted to the ECG examination. ECG was acquired in dogs in a standing position with BTL SD-8 electrocardiographic device and analyzed once the recording was enlarged. P-wave duration was calculated in 9 ECG leads (I, II, III, aVR, aVL, aVF, V1, V2, V4) from 5 cardiac cycles.

**Results:**

The proper P-wave dispersion in healthy dogs was determined at up to 24 ms. P-wave dispersion was statistically significant increased (p < 0.01) in dogs with chronic valvular disease and dogs with disturbances of supraventricular conduction. In dogs with the atrial enlargement the P-wave dispersion is also higher than in healthy dogs, although no significant correlation between the size of left atria and Pd was noticed (p = 0.1, r = 0,17).

**Conclusions:**

The P-wave dispersion is a constant index in healthy dogs, that is why it can be used for evaluating P wave change in dogs with chronic valvular disease and in dogs with disturbances of supraventricular conduction.

## Background

P-wave dispersion (P_d_) is an ECG index evaluated in human cardiology and veterinary medicine [1-3]. The index is defined as the difference between the maximum and minimum P-wave duration recorded from different ECG leads. It is assumed that the duration of the P-wave and the P_d _reflect the electrophysiological properties of the atrium muscle. As the electrical activity of the cardiac muscle displayed on the electrocardiogram is closely correlated with the conduction of specific areas of the atrium; the regional depolarization disturbances may lead to variety of the duration of the P-wave at different ECG leads. Changes in the P_d _may reflect the disturbances in the inter and intra-atrial conduction and the inhomogeneous propagation of the sinus impulses. It is not clearly stated if only the conduction heterogeneity of atria (local effect) or also the various projection of the single depolarization vector at different ECG leads (projection phenomenon) [4,5] will have the influence on the interlead variation of the P-wave duration. Important can be also the obstacle in measurements, when the P-wave amplitude is small and its onset and offset are difficult to determine.

The P_d _is also evaluated in humans as a prognosis index in case of atrial fibrillation (AF) [6-8]. It is assumed, that this way, there will be a possibility to detect patients that do not show visible heart disorders although have a higher risk in developing AF [6,7]. In veterinary medicine, up to now, the P_d _has been evaluated only at healthy dogs to establish the proper values of this index [1]. Many hopes are being placed on using P_d_, as an indicator, in dogs predisposed to develop some types of supra-ventricular arrhythmia, for example AF in dogs, in dogs that are suspected to have dilated cardiomyopathy, in dogs with enlarged atria due to mitral/tricuspid insufficiency or in dogs predisposed to sinus disorders. No research have been fulfilled to evaluate the P_d _in dogs with supra-ventricular conduction disorders or in dogs with enlarged atria.

The aim of this study was to evaluate the P-wave dispersion in healthy dogs, in dogs with mitral valve insufficiency and in dogs with supra-ventricular conduction disorders.

## Methods

The study was performed on 88 dogs, divided into three groups. The first group included 53 dogs (22 females/31 males): 6 German Shepherds, 2 Miniature Pinschers, 3 Yorkshire Terriers, 2 Giant Schnauzers, 2 Shih-tzus, 1 Mastino Napoletano, 8 Mixed breeds, 2 Great Danes, 2 Golden Retrievers, 3 Dachshunds, 1 Irish Setter, 1 Cairn Terrier, 1 Tibetan Mastiff, 2 Rottweilers, 1 Flat Coated Retriever, 2 st. Bernards, 3 American Staffordshire Terriers, 1 Bulmastiff, 2 German Pointers, 1 West Highland White Terrier, 1 Bouvier des Flandres, 1 Beagle, 1 Border Collie, 1 Scottish Terrier, 1 Boxer, 1 Dalmatian, 1 Chinese Crested Dog. The body weights were between 1,5 and 80 kg, aged from 0.5 to 17 years. All dogs did not show abnormalities in clinical examination, ECG and echocardiography (ratio LA/Ao < 1.2)

The second group included 23 dogs with mitral valve insufficiency (5 females/18 males): 1 Shih-tzu 1 Yorkshire Terrier, 1 Miniature Pinscher, 7 Mixed breeds, 8 Dachshunds, 3 Miniature Poodles, 2 Miniature Schnauzers, body weights between 3,3 and 38 kg, aged from 8 to 17 years. All dogs in this group in clinical examination had heart murmurs (level of 3 to 5) and clinical sings of heart failure (Ib, II and IIIa, ISACHC score) [9], mitral valve insufficiency and the enlargement of left atria confirmed in echocardiography and ratio LA/Ao > 1.5. According to Bonagura et al the standard for LA/Ao is 1.2, although in the literature appear values up to 1.5 in healthy dogs [10-12], that is why in this study it was assumed that value LA/Ao >1.5 (group II) indicated the atria enlargement.

Tricuspid valve insufficiency of small degree was noticed at 5 dogs. P_d _was calculated for dogs that were not treated earlier for cardiac disease. Group III contained 12 dogs (5 females/7 males) with supra-ventricular conduction disorders: 1 Mixed breed (sino-atrial block), 1 Great Dane (atrio-ventricular block 1'st degree), 2 Golden Retrievers (atrio-ventricular block 1'st and 2'nd degree), 2 Dachshunds (sino-atrial block, atrio-ventricular block 1'st degree), 1 Beagle (sino-atrial block), 1 Pug (atrio-ventricular block 1'st degree), 2 Miniature Schnauzers (sino-atrial block, atrio-ventricular block 1'st degree) 1 Labrador Retriever (sino-atrial block), 1 Bullmastiff (atrio-ventricular block 2'nd degree), with body weight between 7 and 70 kg and aged from 14 months till 12 years.

The animals were qualified based upon earlier investigation, preliminary clinical examination and morphological and biochemistry blood sampling (AST, ALT, urea, creatinine, Na^+^, K^+^, Ca^2+^, Mg^2+^, Cl^-^). No variations from normal parameters were detected. All dogs went through echocardiography to establish the size of the heart caves and functions of specific structures (contractility of the left ventricle and the function of atrio-ventricle valves). The LA/Ao ratio was obtained by measuring the left atria and aorta diameters in the ventricular's endsystolic ECG phase [11,13]. The echocardiography examination was performed on the echocardiograph ALOKA 4000+. The probe used for echocardiography was sector type 5 MHz and 7.5 MHz.

All dogs underwent ECG in standing position on BTL SD08 equipped with net filter and different frequencies of muscular filters. The ECG signals were recorded as a direct electronic signal every 30 seconds using computer software BTL. Additionally the computer system for ECG record evaluation allows to reduce the interference of muscles on the ECG record and in the same time eliminate those artefacts. The system enables to enlarge the record 200 times while using a computer display 21,3". The electrodes are placed accordingly: right arm (red electrode), left arm (yellow electrode), right leg (black electrode) and left leg (green electrode). The precordial leads were attached as follows: V1 was placed right of sternum at the 5^th ^intercostal space, V2 - was placed just to the left of the sternum, V4 - was placed to the left at the costochondrial junction at the 6^th ^intercostal space [14]. The record was analyzed carefully to calculate the P-wave dispersion. The evaluation of P-wave duration was done on 9 ECG leads (I, II, III, IV, aVR, aVL, aVF, V_1_, V_2_, V_4_) at five cardiac cycles. The assessment was done by the means of electronic markers on the computer screen after a 200 times enlargement of the ECG record. In every evaluated lead the duration of P-wave was measured as a distance between the onset (positive or negative deflection from the isoelectric line) and the offset (return to the isoelectric line) with precision to 1 ms. After that, minimum (P_min_) and maximum (P_max_) values of P-wave was set. The dispersion of P-wave was calculated as the difference between P_max _and P_min _and then the average from 5 measurements have been obtained.

When the electrocardiography measurements were completed all data were subjected to statistical analysis. The deviation between values of P_d _were analyzed based on Mann-Whitney U test and the correlation between the objective index of atria's size (the size of the left atria compared to the size of aorta - LA/Ao) and P_d _was evaluated. We carry out multiple linear regression dependence of P_d _from body mass, age, sex and LA/Ao. Statistical analysis was based on program STATISTICA, version 7.1.

The studies obtained consent of the 2nd Local Ethical Commission No 06/2008.

## Results

**Figure 1:**
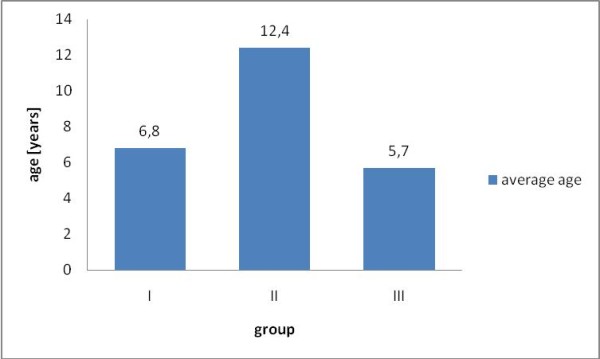
**Avarage age of the dogs in particular groups**. Statistical significant difference (p < 0.05) between group II (n = 23) and groups I (n = 53) and III (n = 12).

**Figure 2:**
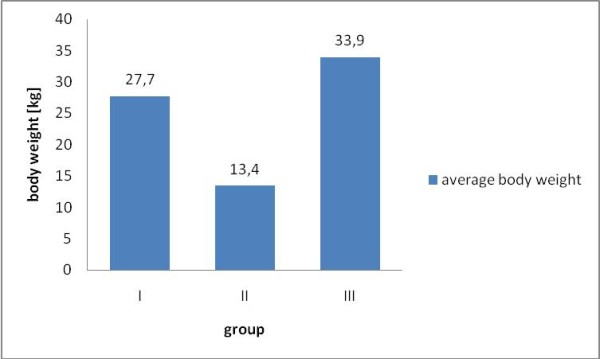
**Average body mass of the dogs in particular groups**. Statistical significant difference (p < 0.05) between group II (n = 23) and groups I (n = 53) and III (n = 12).

Figure 1 shows the average age of the dogs in particular groups. The age is significantly higher at dogs showing a degeneration of the mitral valve (p < 0.05). The average weight of the dogs in particular groups is shown in Figure 2 - dogs having the degeneration of mitral valve (CVD) have lower body weight than the dogs in other groups (p < 0.05).

In all evaluated groups of dogs there were more males then females.

Based upon the results from group I the mean values for P_max_, P_min_, P_d_, were calculated which were accordingly: P_max_- 63.4 ± 12.7 ms, P_min_- 46.6 ± 11.5 ms, P_d_- 16.8 ± 3.51 ms (range 9.2-22.6 ms, dominant = 16.2). The correct value of P-wave dispersion of healthy dogs was set as a mean value of P_d _± 2SD and it was less than 24 ms.

Based on values received from healthy dogs, the dependency of P_d _from other parameters such as: body weight (table 1), age (table 2) and sex (table 3) were analyzed. No significant deviation of P_d _was noticed according to body weight, age, and sex.

**Table 1:** P_d _in healthy dogs depending on the body mass

Body weight	P_min _[ms]	P_max _[ms]	P_d _[ms]	SD
<10 kg	36,3	52,0	15,7	4,7

10-30 kg	48,2	65,6	17,5	3,6

>30 kg	51,6	68,7	17,0	3,6

P_min _= the minimum duration of P wave, P_max _= maximum duration of P wave, P_d _= P wave dispersion

**Table 2:** P_d _in healthy dogs depending on the age

Age	P_min _[ms]	P_max _[ms]	P_d _[ms]	SD
<2 years	48,3	65,3	17,0	3,7

2-8 years	45,7	63	17,3	2,9

>8 years	44,3	60,5	16,2	4,6

P_min _= the minimum duration of P wave, P_max _= maximum duration of P wave, P_d _= P wave dispersion

**Table 3:** P_d _in healthy dogs depending on the sex

Sex	P_min _[ms]	P_max _[ms]	P_d _[ms]	SD
Male	47,4	64,2	17,1	4

Female	45,3	62	16,4	3,9

P_min _= the minimum duration of P wave, P_max _= maximum duration of P wave, P_d _= P wave dispersion

**Figure 3:**
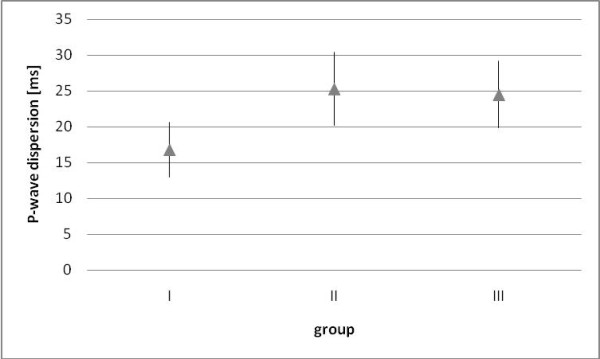
**Average P wave dispersion (mean ± SD) in particular groups**. Statistical significant difference (p < 0.01) between group I (n = 53) and groups II (n = 23) and III (n = 12).

The results (P_d_) were compared between healthy dogs (group I), dogs with mitral valve insufficiency (group II) and dogs with supra-ventricular conduction disorders (group III). Dogs with mitral valve insufficiency and dogs with supra-ventricular conduction disorders had significantly higher values of P_d _than healthy dogs (p < 0.01) (Figure 3). The received results were also higher than proposed norm (mean value ± SD) and were accordingly: 25,3 ± 5,1 ms (range 19.2-30.8) in group II and 24.5 ± 4.7 ms (range 15.2-30.9) in group III (table 3). The dependency of P_d _from the level of left atria enlargement were also analyzed, such as the correlation between P_d _and the LA/Ao ratio coefficient. Statistically the dispersion of P-wave did not differ (p = 0.86) between groups of dogs having visible enlargement of left atria (LA/Ao 2.2 ± 1.3) or disorders of supra-ventricular conduction (LA/Ao 1.4 ± 0.6). In the group of dogs with insufficiency of mitral valve there were no correlations noticed with the increase of P_d _and the level of left atria enlargement (p = 0.1, r = 0.17). In multiple linear regression dependence of P_d _from body mass, age, sex and LA/Ao was controlled, and only P_d _is an independent parameter in the multiple linear regression.

## Discussion

In veterinary electrocardiography the gold standard is to perform the ECG record in a recumbence, nether less literature shows that the ECG record can be performed also in a standing or sternum position. In many publications the ECG records were compared coming from dogs that were in lateral recumbency or standing position. It has been noticed that the position of the dog does not influence the P-wave duration, P-wave amplitude or PR interval [15,16]. Dogs standing position is used also during toxicological and pharmacological examination. In the Hanton and Rabamampiania study it is stated that the body position of the dog during recording of ECG had no major influence on most parameters. In many studies taking up the problem of the influence of the dog's position on the ECG record parameters the sufficient impact on the modified mean electrical axis is underlined, what was not evaluated in this research study [15-18]. Standing position was chosen in this study due to lack of any documented muscle interferences with duration and amplitude of P-wave and duration of PR interval, lower stress for the animal and that means less heart rhythm frequency.

The frequency and degree of degeneration of atrio-ventricular valves increases in older dogs. That is why the dogs that had the insufficiency of mitral valve were, in average, the older ones [19]. Degeneration progress with age. In the same group of dogs the body weight was lower, which is correlated with the predisposition of smaller and miniature breeds to the degeneration of mitral valve [19]. No dependency between the age of healthy dogs and P_d _was noticed. A tendency for greater spread of P_d _was observed more often in healthy dogs above 8 years old, that was pictured by the P_d _standard deviation increase (table 2). There is no correlation between the body weight and P_d _in healthy dogs. There is an increase of the average maximum and minimum duration of P-wave, correlated with the increase of body weight, which goes together with the increased size of the heart, particularly the size of atrias. The ratio of these values is constant, so there are no statistical differences between particular body weight groups of healthy dogs. No correlation between sex and the value of P_d _was noticed, even though there were more males than females. The appearance of higher number of males is due to preferences of the owners to have male dogs, not due to the correlation between sex and heart disorders. Received values of P_d _in healthy dogs, independently from age, body weight and sex had small dispersion and small standard deviation. The maximum value of P_d _in this group of dogs was 20.8 ms and was lower than average P_d _values in the group of dogs with mitral insufficiency (group I) and dogs with supra-ventricular conduction disorders (group III).

Presented data allow to assume that P_d _value is an independent factor from body weight and sex. It is a constant parameter in healthy dogs, with no supra-ventricular conduction disturbances and changes in the atria size, resulting in low SD value in a big and diversified group of healthy dogs. This allows to use the P_d _value as an independent parameter for evaluating inter and intra-atrial conduction.

There was noticed a significant increase in P_d _in dogs with increased left atria due to insufficiency of the mitral valve compared to healthy dogs. Mitral valve insufficiency is a complex pathological process, in which takes part, in example, the degeneration of collagen. Acid mucopolisaccharides group around the petals of the valves which at results in nodular thickening, deformation and weakening of the petals which leads to valve insufficiency. Valve insufficiency leads to the enlargement of the belonging atria, anulus fibrosus and ventricle. In the atria appears endocardial and atria muscular fibrosis, intraparietal infarcts and changes in the arterial vessels caused by the stream of regurgitation over the insufficient valve. These processes lead to inhomogeneous propagation of the impulses in the atria which together with the enlargement of the atria impacts the increase of P-wave dispersion. It seems that Pd is more dependant from disturbances of inter and intra-ventricular conduction and inhomogeneous propagation of impulses, than from the level of left atria enlargement. Correlation wasn't noticed between P_d _and the level of the enlargement of left atria. Similar results were found in humans with hypertension, who had earlier episodes of AF or at those that had attacks of AF shortly after P_d _measurements. In these tests no correlation has been noticed between the value of blood pressure, size of left atria and weight of left atria [8,19-21]. Statistically significant increase in P_d _was observed in a group of dogs with supra-ventricular conduction disorders compared to healthy dogs. Average value of P_d _was the highest in this group of dogs, which is directly correlated with improper atria conduction. The duration of P and P_d _is dependant not only from disorders in the atrium. Anemia and activity of immune system can also lead to changes in the auriculars and speed of impulse conduction [22,23]. Infarct, dilatated cardiomyopathy, stricture of the left atrioventricular ostium opening or congenital malformations of the heart can also lead to increased dispersion of P-valve [23-28]. In human medicine the peculiarity and sensitivity of P_d _has been proved and is used as a parameter allowing to detect patients with higher risk of occurring or with the recurrence of atrial fibrillation [6-8]. Presented results, in this study, may also contribute to propagation of similar using values of P-wave dispersion for dogs, but it still demands further research. There are other factors, that can influence P_d_, that should be taken under consideration when interpreting the results. P_d _can increase also during endocrinology disorders such as diabetes and thyroid hyperfunction [29-32], but also at patients that have the terminal phase of renal failure [33,34]. The changes in P_d _have been noticed also in connection with changes of the tension of autonomous nervous system, for example while conducting Valsalva maneuver [35,36] or in connection with panic attacks [37]. That is why it is important to interpret P_d _in connection with other examination results and general overview of the patient.

## Conclusions

P-wave dispersion is a constant parameter in healthy dogs, independent from body weight, age and sex. In dogs with inter and intra-atrial conduction disturbances P-wave dispersion is significantly higher, that is why this parameter can be used to evaluate the possibility of the inter and intra-atrial conduction disturbances. In dogs with chronic valvular disease and the atrial enlargement the P-wave dispersion is also higher than in healthy dogs, although no significant correlation between the size of left atria and Pd was noticed. The dependency with association between of inter and intra-atrial conduction disturbances with Pd in this group of dogs demands further studies.

## Competing interests

The authors declare that they have no competing interests.

## Authors' contributions

ANN planned the study, carried out ECG and echocardiographic examinations, calculated Pd and drafted the manuscript. AS calculated Pd. UP carried out echocardiographic examinations. JN drafted the manuscript. All authors read and approved the final manuscript.
